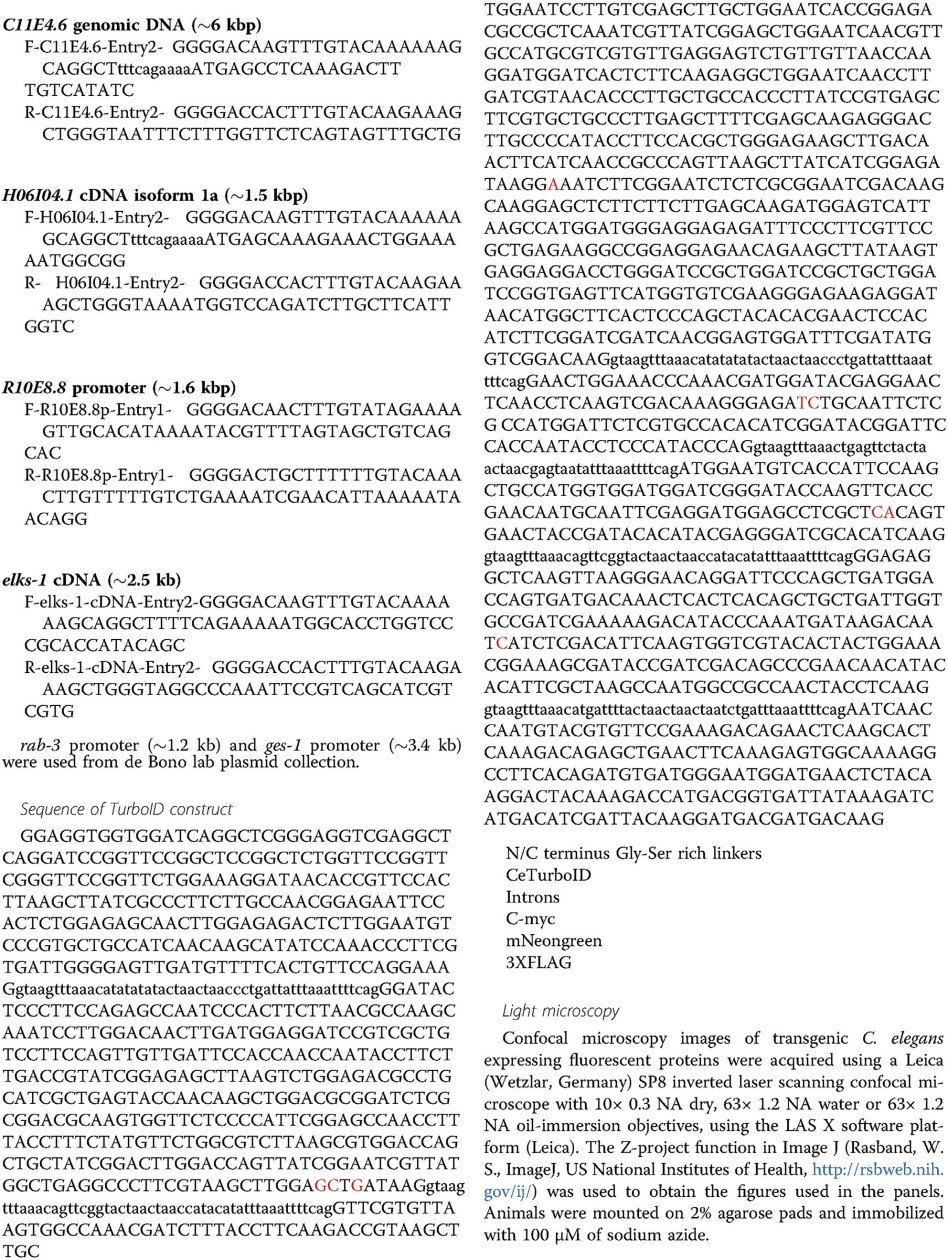# Correction: Interactome analysis of *Caenorhabditis elegans* synapses by TurboID-based proximity labeling

**DOI:** 10.1016/j.jbc.2022.102081

**Published:** 2022-06-01

**Authors:** Murat Artan, Stephen Barratt, Sean M. Flynn, Farida Begum, Mark Skehel, Armel Nicolas, Mario de Bono

During the typesetting of the article above an error was introduced that resulted in 9 missing nucleotides on page 10. Please see the corrections, which are set in red in Figure below. Production apologizes for the error.

**Figure:**