# Supplementary material for: Interactome analysis of Caenorhabditis elegans synapses by TurboID-based proximity labeling
Source: J Biol Chem. 2021 Aug 18;297(3):101094. doi: 10.1016/j.jbc.2021.101094 (PMC8446793; doi:10.1016/j.jbc.2021.101094)
Supplement: Figures S1–S4 [file mmc3.pdf]

Supplementary Materials for

Interactome analysis of *C. elegans* synapses by TurboID-based proximity labeling

Murat Artan[1,2], Stephen Barratt[1,2], Sean M. Flynn[1,3], *Farida Begum*[1], Mark Skehel[1], Armel Nicolas[2], Mario de Bono[1,2,*]

[*]Corresponding author. E-mail: mdebono@ist.ac.at

This pdf file includes:

Figures S1 to S4 and Figure legends

Table S1 and S2 legends

Figure S1

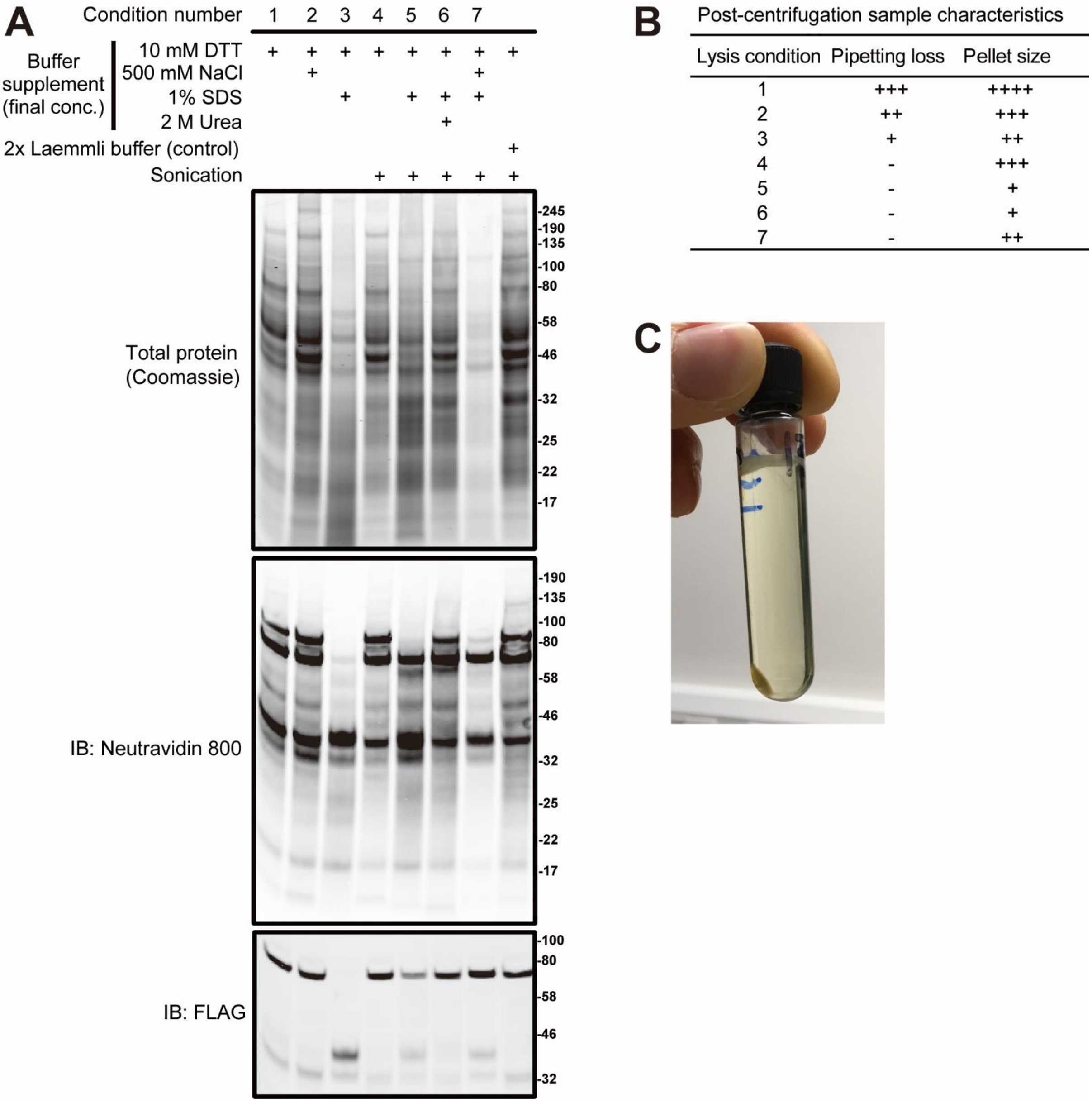

Figure S1. Optimizing extraction and solubilization of *C. elegans* proteins. (**A**) Different extraction protocols compared by visualizing total protein (Coomassie-stained gel, top), biotinylated protein (Western blot probed with Neutravidin 800), and the FLAG-tagged TurboID fusion (Western blot probed with anti-FLAG antibody). Tissue samples were from worms expressing pan-neuronal free TurboID (*rab-3p::TbID::mNG*). We used Coomassie stain intensity at high molecular weights as a

measure of solubilisation efficiency and of limited protein degradation during lysis. Condition 6 was most similar to the solubilisation obtained using 2x Laemmli protein sample buffer, the gold standard, likely as a result of the aggressive denaturation with SDS and the chaotrope urea.

(**B**) Post-centrifugation pellet sizes obtained with the 7 extraction conditions in (A). The compactness of the post-ultracentrifugation pellet and the nature of the attendant lipidic over-phase can affect the protein yield through pipetting loss. Incomplete solubilisation, or incomplete DNA shearing, results in either a large unstable pellet or a substantial turbid lipid layer in the centrifuge tube. The denaturing lysis protocol enabled clarification by ultracentrifugation at room temperature, which improves lipid solubility. Extensive sonication sheared sample DNA and disrupted aggregated material to yield near-complete solubilisation. The optimized protocol allowed us to aspirate ~ 90% of the solubilised sample for subsequent steps. In less optimal conditions up to 50% of the sample by volume could be lost at the post-centrifugation aspiration step as we tried to avoid transferring suspended insoluble material. Ensuring a high degree of clarification in the post-centrifugation soluble fraction was important to limit non-specific bead binding by carried-over insoluble material.

(**C**) Typical pellet appearance following clarification by ultracentrifugation following selected extraction method showing >=95% solubilization by volume. Tube capacity in the image is 10.4 ml.

Figure S2

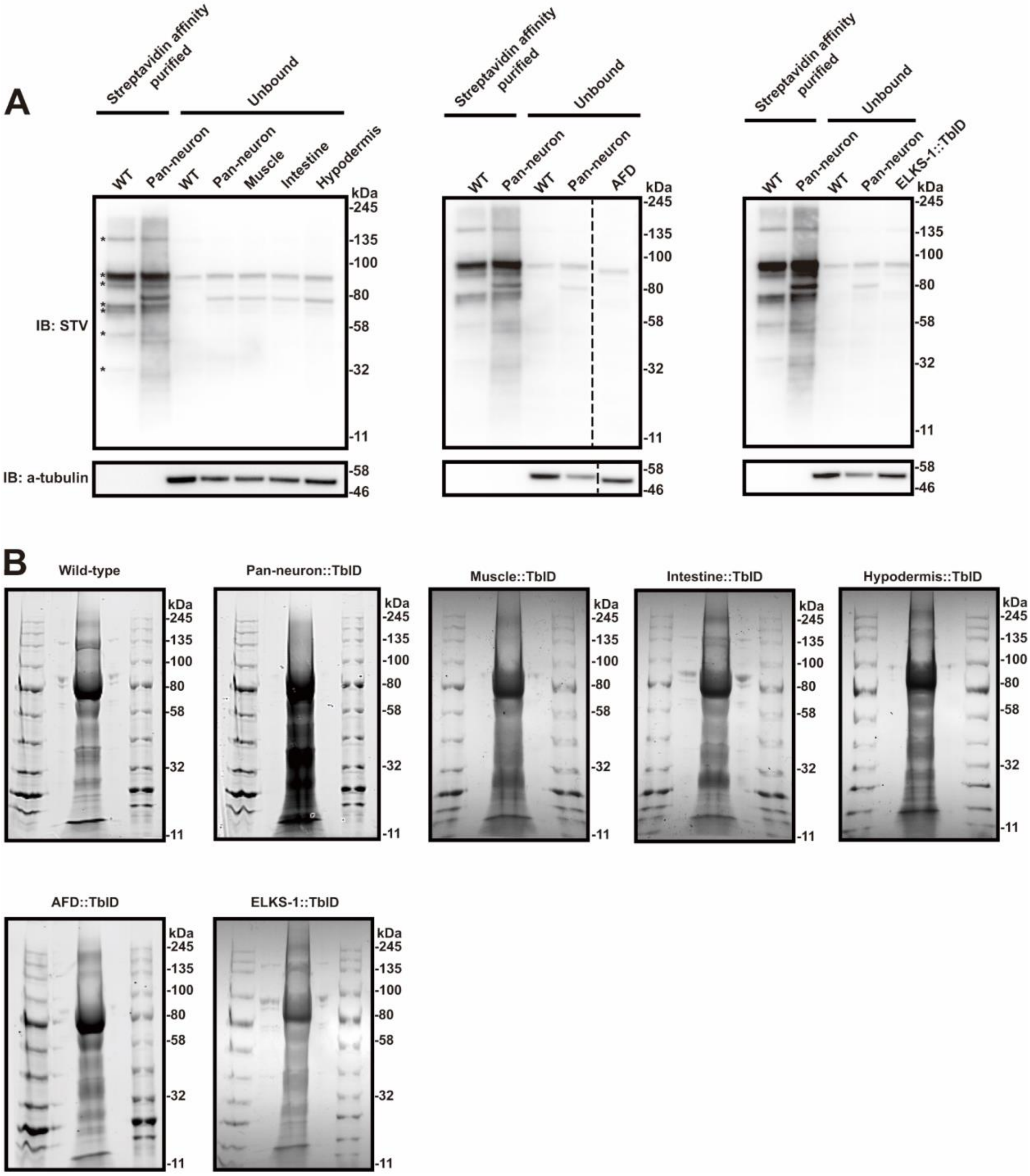

Figure S2. Testing the efficiency of streptavidin affinity purification of biotinylated proteins. (**A**) Western blot analysis of bound and unbound biotinylated proteins after streptavidin-based affinity purification of samples. (**B**) Proteins isolated from various samples and affinity purified using streptavidin beads, separated using 4-12% Bis-

Tris gels and stained by Coomassie. Also shown are molecular weight markers.

Slices from these gels were sent for mass spectrometry.

Figure S3

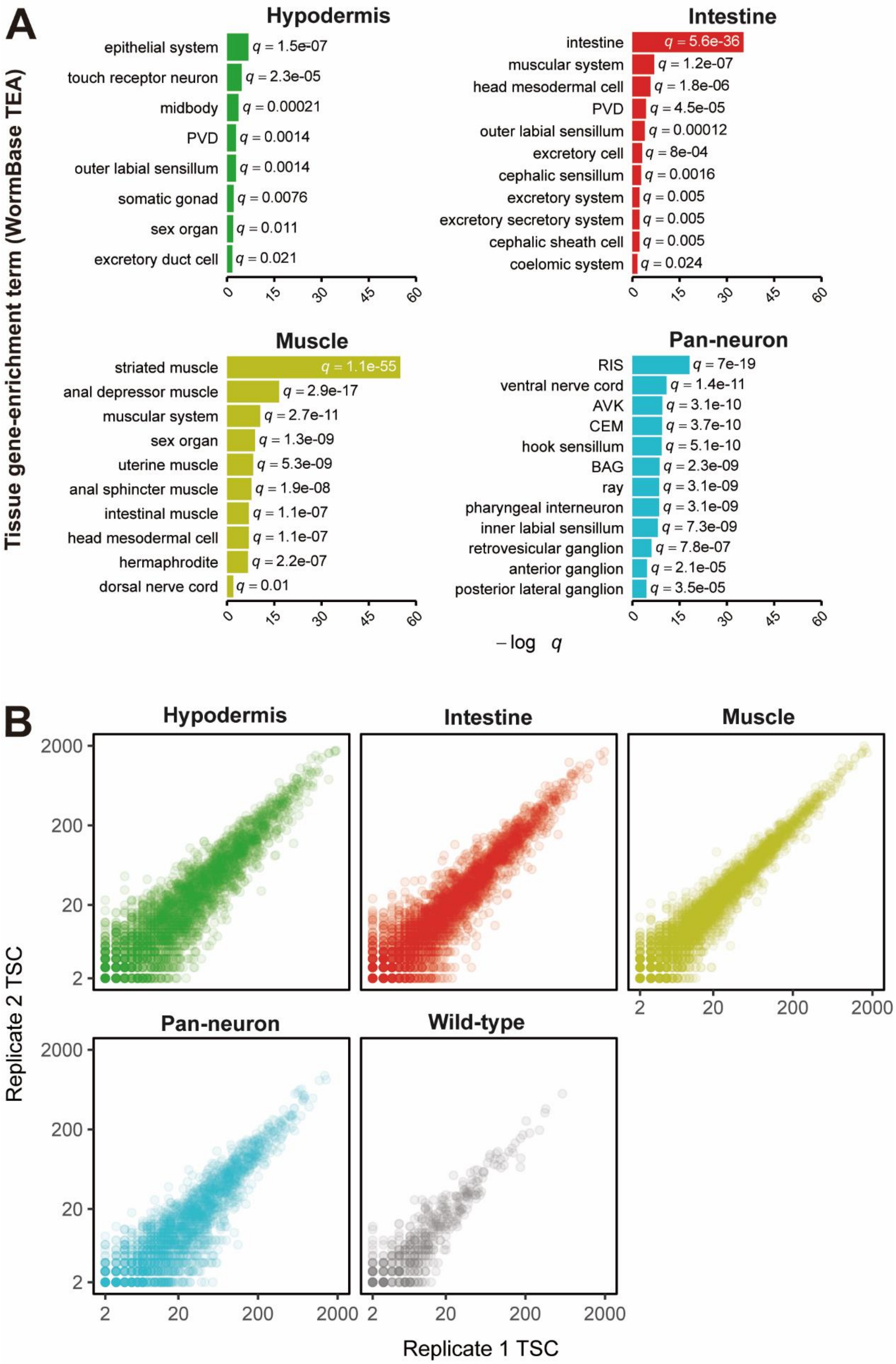
A
Tissue gene-enrichment term (WormBase TEA)
Hypodermis
epithelial system q = 1.5e-07
touch receptor neuron q = 2.3e-05
midbody q = 0.00021
PVD q = 0.0014
outer labial sensillum q = 0.0014
somatic gonad q = 0.0076
sex organ q = 0.011
excretory duct cell q = 0.021
Intestine
intestine q = 5.6e-36
muscular system q = 1.2e-07
head mesodermal cell q = 1.8e-06
PVD q = 4.5e-05
outer labial sensillum q = 0.00012
excretory cell q = 8e-04
cephalic sensillum q = 0.0016
excretory system q = 0.005
excretory secretory system q = 0.005
cephalic sheath cell q = 0.005
coelomic system q = 0.024
Muscle
striated muscle q = 1.1e-55
anal depressor muscle q = 2.9e-17
muscular system q = 2.7e-11
sex organ q = 1.3e-09
uterine muscle q = 5.3e-09
anal sphincter muscle q = 1.9e-08
intestinal muscle q = 1.1e-07
head mesodermal cell q = 1.1e-07
hermaphrodite q = 2.2e-07
dorsal nerve cord q = 0.01
Pan-neuron
RIS q = 7e-19
ventral nerve cord q = 1.4e-11
AVK q = 3.1e-10
CEM q = 3.7e-10
hook sensillum q = 5.1e-10
BAG q = 2.3e-09
ray q = 3.1e-09
pharyngeal interneuron q = 3.1e-09
inner labial sensillum q = 7.3e-09
retrovesicular ganglion q = 7.8e-07
anterior ganglion q = 2.1e-05
posterior lateral ganglion q = 3.5e-05
0 15 30 45 60
– log q
B
Hypodermis
Intestine
Muscle
Pan-neuron
Wild-type
Replicate 2 TSC
Replicate 1 TSC
2 20 200 2000

Figure S3. Tissue enrichment analysis of unique protein hits identified by mass spectrometry analysis of four major *C. elegans* tissues. (**A**) Top-ranked Wormbase tissue-enrichment analysis (TEA) terms associated with tissue-enriched protein hits detected across indicated samples. (**B**) Replicate 1 vs replicate 2 total spectral counts for protein hits from indicated samples. Points correspond to individual proteins identified.

Figure S4

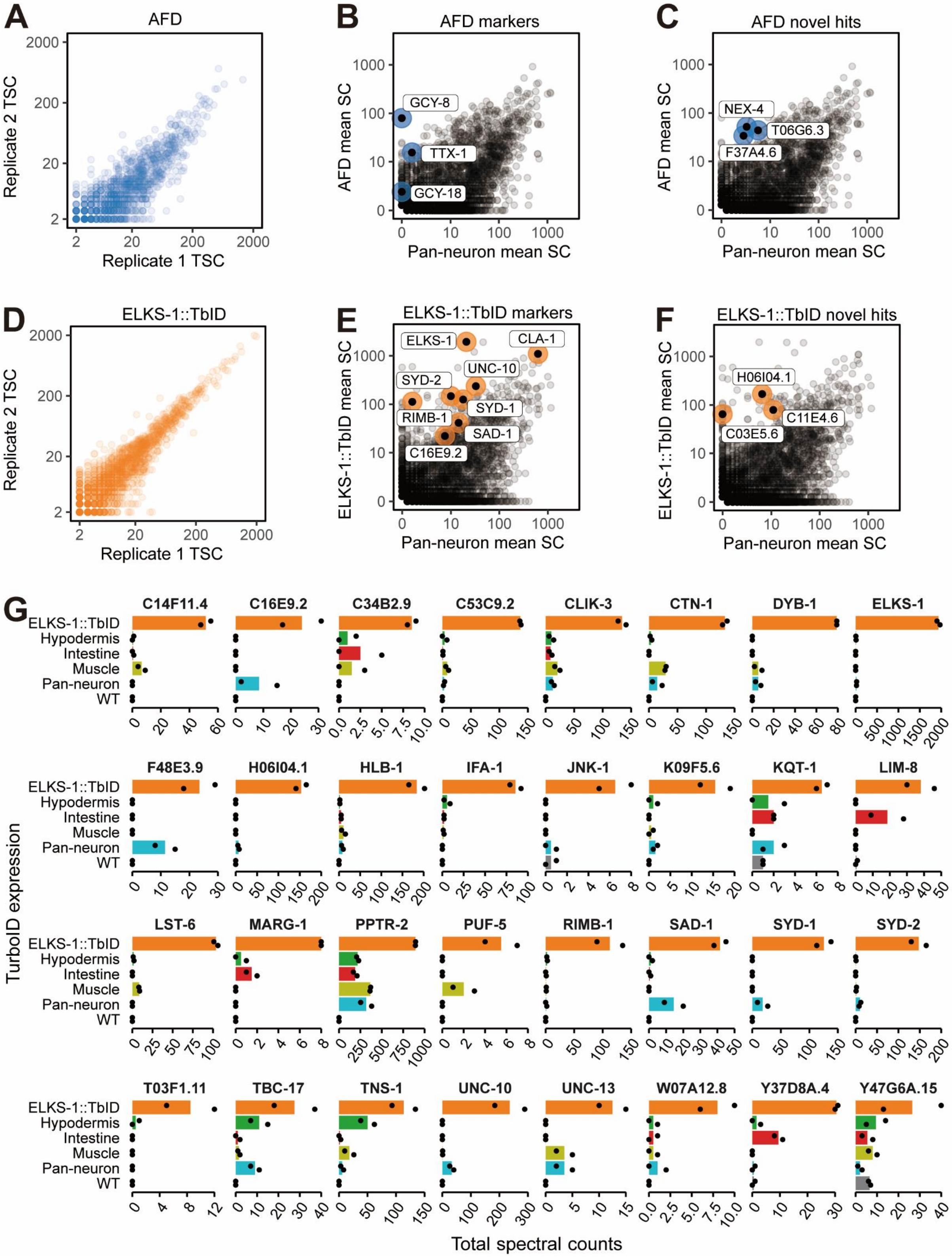

A
AFD
Replicate 2 TSC
Replicate 1 TSC
B
AFD markers
GCY-8
TTX-1
GCY-18
AFD mean SC
Pan-neuron mean SC
C
AFD novel hits
NEX-4
T06G6.3
F37A4.6
AFD mean SC
Pan-neuron mean SC
D
ELKS-1::TbID
Replicate 2 TSC
Replicate 1 TSC
E
ELKS-1::TbID markers
ELKS-1
CLA-1
UNC-10
SYD-2
SYD-1
RIMB-1
SAD-1
C16E9.2
ELKS-1::TbID mean SC
Pan-neuron mean SC
F
ELKS-1::TbID novel hits
H06I04.1
C11E4.6
C03E5.6
ELKS-1::TbID mean SC
Pan-neuron mean SC
G
C14F11.4
C16E9.2
C34B2.9
C53C9.2
CLIK-3
CTN-1
DYB-1
ELKS-1
F48E3.9
H06I04.1
HLB-1
IFA-1
JNK-1
K09F5.6
KQT-1
LIM-8
LST-6
MARG-1
PPTR-2
PUF-5
RIMB-1
SAD-1
SYD-1
SYD-2
T03F1.11
TBC-17
TNS-1
UNC-10
UNC-13
W07A12.8
Y37D8A.4
Y47G6A.15
ELKS-1::TbID
Hypodermis
Intestine
Muscle
Pan-neuron
WT
TurboID expression
Total spectral counts

Figure S4. Spectral counts analysis of proteins across replicate samples of AFD-specific TurboID and ELKS-1::TurboID. (**A**) Replicate 1 vs replicate 2 total protein counts for protein hits. Points correspond to individual proteins identified in AFD neurons. (**B** and **C**) Comparison of protein mean spectral counts between AFD and pan-neuronal free TurboID samples. Known marker proteins (**B**) and novel hits (**C**) are highlighted. (**D**) Replicate 1 vs replicate 2 total protein counts for protein hits. Points correspond to individual proteins identified in ELKS-1::TurboID. (**E** and **F**) Comparison of protein mean spectral counts between ELKS-1::TurboID and pan-neuronal free TurboID samples. Known marker proteins (**E**) and novel hits (**F**) are highlighted. (**G**) Proteins enriched two fold or more in ELKS-1::TurboID samples compared to other samples, and represented by a mean of at least 5 spectral counts.

Supplemental table legends

Table S1: Total spectral counts of protein hits identified in tissues, AFD and ELKS-1::TbID samples.

Table S2: Distinct peptides identified and protein coverage for mass spectrometry analyses in tissues, AFD and ELKS-1::TbID samples.
